# Occult Late-Onset Systemic Lupus Erythematosus Presenting as Critical Multisystem Disease in a Male: A Diagnostic Challenge in the Intensive Care Unit

**DOI:** 10.7759/cureus.114919

**Published:** 2026-08-21

**Authors:** Md Shamsuzzaman, Ahmed Eldesoky, Ramy Mosallam, Nuoma Munir, Pradeep Sapkota

**Affiliations:** 1 Acute Internal Medicine, Mid and South Essex NHS Foundation Trust, Essex, GBR; 2 Critical Care, Mid and South Essex NHS Foundation Trust, Essex, GBR

**Keywords:** critical multisystem disase, diagnostic and therapeutic challenge, intensive care unit, late-onset systemic lupus erythematosus, subacute cutaneous lupus erythematosus (scle)

## Abstract

Systemic lupus erythematosus (SLE) is a heterogeneous autoimmune disease that predominantly affects women of reproductive age. Late-onset SLE in males is uncommon and frequently presents with atypical clinical features, often resulting in diagnostic delay. We report a 60-year-old male admitted to the intensive care unit (ICU) with melena and haemodynamic instability on a background of a recent non-ST-elevation myocardial infarction (NSTEMI). During investigation, severe esophageal candidiasis, bilateral pleural effusions, proteinuria, microscopic haematuria, and widespread cutaneous lesions were identified. Extensive infectious and malignancy screening was negative. Autoimmune testing demonstrated strongly positive antinuclear antibodies (ANA), high-titre anti-double-stranded DNA antibodies (anti-dsDNA), positive extractable nuclear antigen (ENA) profile, hypocomplementemia, and lupus anticoagulant positivity. Skin biopsy was suggestive of subacute cutaneous lupus erythematosus (SCLE). The patient fulfilled the 2019 European Alliance of Associations for Rheumatology/American College of Rheumatology (EULAR/ACR) classification criteria for SLE. Treatment with pulse intravenous methylprednisolone followed by oral corticosteroids and hydroxychloroquine resulted in significant clinical improvement. This case highlights the diagnostic complexity of late-onset male SLE presenting with critical multisystem disease in the ICU and emphasises the importance of considering autoimmune disease in patients with unexplained multisystem inflammatory manifestations.

## Introduction

Systemic lupus erythematosus (SLE) is a complex autoimmune disorder characterised by multisystem inflammatory involvement and highly variable clinical manifestations. The disease predominantly affects women of reproductive age, with female-to-male ratios approaching 9:1 in younger populations [1]. However, late-onset SLE is typically defined as disease onset after the age of 50 and accounts for approximately 10% to 20% of lupus cases reported in the literature [2]. Compared with younger patients, those with late-onset disease demonstrate distinct clinical and serological characteristics. They tend to exhibit lower frequencies of malar rash and severe nephritis, but increased rates of serositis, pulmonary involvement, and damage accrual [2,3]. The female-to-male ratio also declines with advancing age, reaching closer to 4:1 in older cohorts [3]. Because of these atypical presentations and a generally lower index of clinical suspicion in elderly males, diagnosis is often significantly delayed [2]. We report a diagnostically challenging case of occult late-onset SLE presenting with multisystem disease requiring ICU admission.

## Case presentation

A 60-year-old male presented with melena, fatigue, and haemodynamic instability. His recent history was significant for a non-ST-elevation myocardial infarction (NSTEMI), with coronary angiography demonstrating distal posterior descending artery disease, managed conservatively. He was discharged on dual antiplatelet therapy (aspirin and clopidogrel), proton pump inhibitor, beta-blocker, angiotensin-converting enzyme inhibitor and high-dose atorvastatin.

There was no prior history of immunosuppressive therapy or known immunodeficiency. The patient was an occasional smoker, consuming a few cigarettes per day, and reported intermittent alcohol intake. Family history was notable for his mother having been diagnosed with Miller Fisher syndrome at the age of 80 years.

His initial blood tests on admission were noted for raised CRP (149 mg/L) coupled with dropped haemoglobin to 77 g/l; however, platelet count was 308 along with chronic lymphopenia, and coagulation profile was reported unremarkable with prothrombin time (PT) 12.7 sec, international normalized ratio (INR) 1.1, activated partial thromboplastin time (APTT) 29.5 sec. Due to haemodynamic instability, he was admitted to the intensive care unit for vasopressor support and evaluation of suspected upper gastrointestinal bleeding. Esophagogastroduodenoscopy revealed endoscopic appearance of severe esophageal candidiasis and erosive duodenitis with high risk of re-bleeding. However, CT angiography of the renal arteries and abdomen did not demonstrate any active bleeding point along the visualised portion of the gastrointestinal tract. His upper gastrointestinal bleeding was presumed to be gastric erosion related to a background of aspirin-clopidogrel (DAPT) therapy for recent NSTEMI. He was initially managed with IV proton pump inhibitor, 4 units of red blood cell transfusion and supportive critical care. Aspirin and clopidogrel were suspended temporarily, and following discussion with the gastroenterologist, clopidogrel was resumed as there was no active bleeding.

Given the severity of opportunistic fungal infection, an underlying immunodeficiency was suspected. However, comprehensive infectious workup, including blood cultures, T-SPOT TB (Oxford Immunotec, UK), and viral screening including HIV, hepatitis B, hepatitis C, cytomegalovirus, and Epstein-Barr virus, was negative. Computed tomography of the chest, abdomen, and pelvis together with tumour marker screening demonstrated no evidence of malignancy.

During ICU admission, examination revealed widespread erythematous annular and papulosquamous skin lesions (Figure 1). Importantly, the patient reported that the rash had been present for several weeks before the myocardial infarction and prior to the event of recent NSTEMI, he was on selective serotonin receptor inhibitors (SSRIs) for depressive illness. He subsequently developed progressive dyspnoea. Bedside ultrasonography and computed tomography demonstrated bilateral pleural effusions with atelectatic changes (Figure 2). Pleural fluid analysis revealed an exudative effusion with negative cultures and cytology. Focused echocardiogram showed ejection fraction (EF) 50-55% with grade 1 diastolic dysfunction.

**Figure 1:**
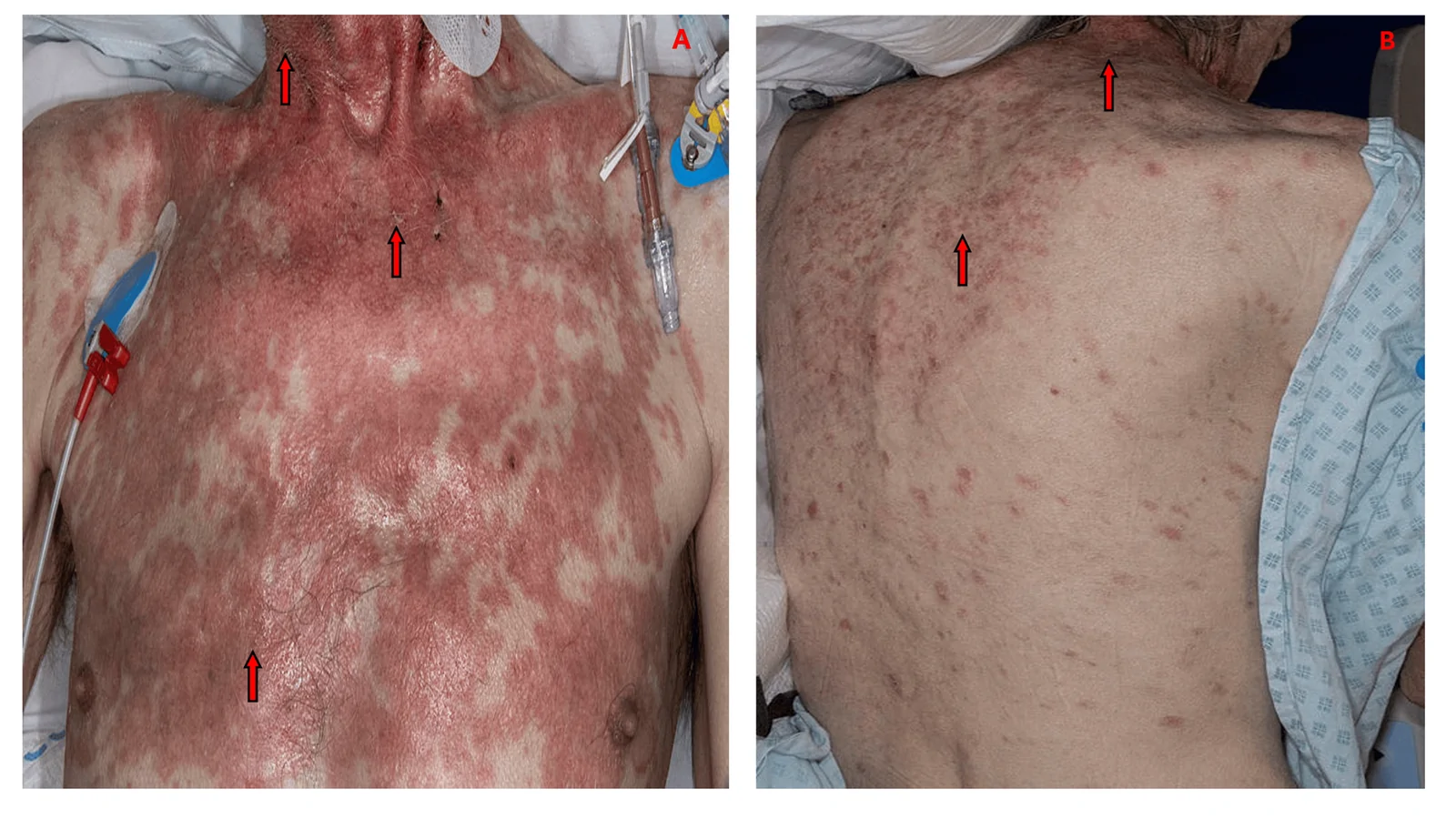
Photodistributed erythematous cutaneous eruption consistent with SCLE: (A) Chest and anterior neck; (B) Upper back and posterior neck. SCLE: Subacute cutaneous lupus erythematosus

**Figure 2:**
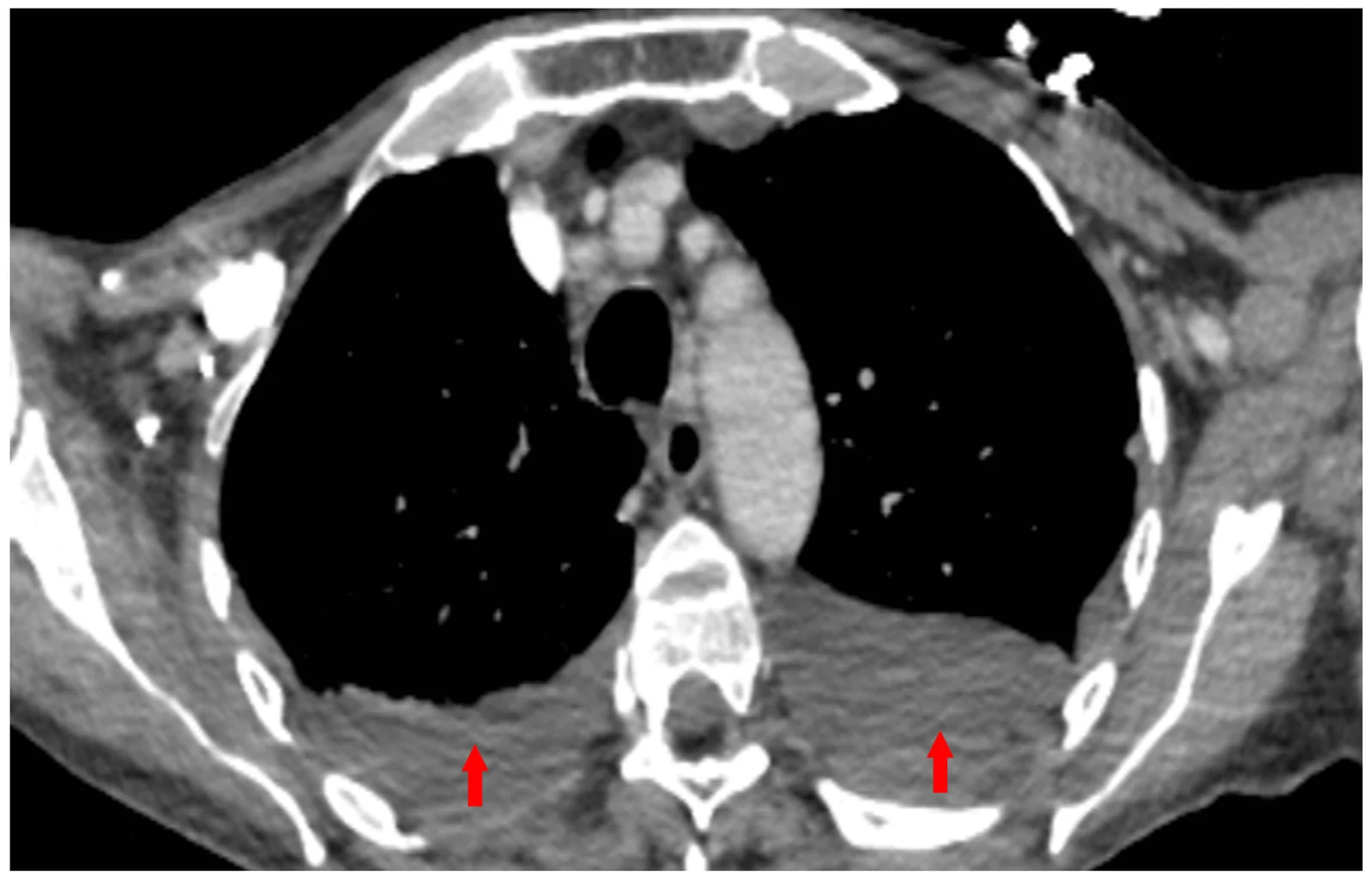
CT of the thorax demonstrated bilateral pleural effusions with associated compressive atelectasis.

Further laboratory investigations demonstrated gradual derangement of liver function tests with alkaline phosphatase (ALP) 749 U/L, alanine aminotransferase (ALT) 177 U/L, and albumin 24 g/L; however, bilirubin and INR were within the normal range. Ultrasound and CT scan of the abdomen did not show any focal liver lesion or biliary dilatation. Additionally, magnetic resonance cholangiopancreatography (MRCP) was also reported as normal. Urine analysis demonstrated culture-negative microscopic haematuria without any casts. Significant urinary protein excretion [urine total protein (UTP) 0.68 g/L, protein creatinine ratio (PCR) 194.3 mg/mmol, albumin creatinine ratio (ACR) 6.29 mg/mmol along with stable creatinine at 34 umol/l)] was noted. He was non-diabetic and reported an HbA1c level of 36 mmol/mol.

An autoimmune serology panel revealed strongly positive antinuclear antibodies (titre 19.2, speckled pattern >1:640 and homogenous pattern 1:160), high-titre anti-double-stranded DNA antibodies (25.8 IU), a positive extractable nuclear antigen profile (48.9) with positive anti-SSA, anti-SSB and anti-Ro52, hypocomplementaemia (C3 0.80, C4 0.13), and lupus anticoagulant positivity. Skin biopsy findings were suggestive of subacute cutaneous lupus erythematosus with a differential of erythema multiforme.

The patient's 2019 EULAR/ACR classification criteria score was 26 for systemic lupus erythematosus [4]. Multidisciplinary review concluded a diagnosis of late-onset SLE with cutaneous and possible pulmonary-renal involvement.

The patient was commenced on intravenous pulse methylprednisolone 500 mg daily for three consecutive days, followed by oral prednisolone 40 mg with a plan for gradual tapering. Given the prominent cutaneous manifestations of possible subacute cutaneous lupus erythematosus, hydroxychloroquine was initiated as disease-modifying therapy. Antifungal treatment with fluconazole 200 mg once a day for three weeks, empirical antibiotics, and supportive ICU care were continued. Escalation to additional disease-modifying antirheumatic drugs was deferred because of concerns regarding severe esophageal candidiasis and deranged liver function tests.

The patient responded well to the treatment. Near-complete resolution of the cutaneous eruption was observed over a few weeks of inpatient hospital stay (Figure 3), accompanied by an improvement in respiratory symptoms and stabilisation of haemodynamic parameters. Renal function remained preserved, and no further progression of urinary abnormalities was observed during admission. Following clinical improvement, he was stepped down from the intensive care unit to the medical ward for ongoing management under the rheumatology and renal teams. Arrangements were made for outpatient follow-up, monitoring of disease activity, and further assessment of suspected lupus nephritis. On subsequent renal follow-up, normalisation of proteinuria along with stable kidney function was noted. He has been scheduled for ongoing follow-up with the rheumatology team, with hydroxychloroquine as an ongoing disease-modifying anti-rheumatic drug (DMARD).

**Figure 3:**
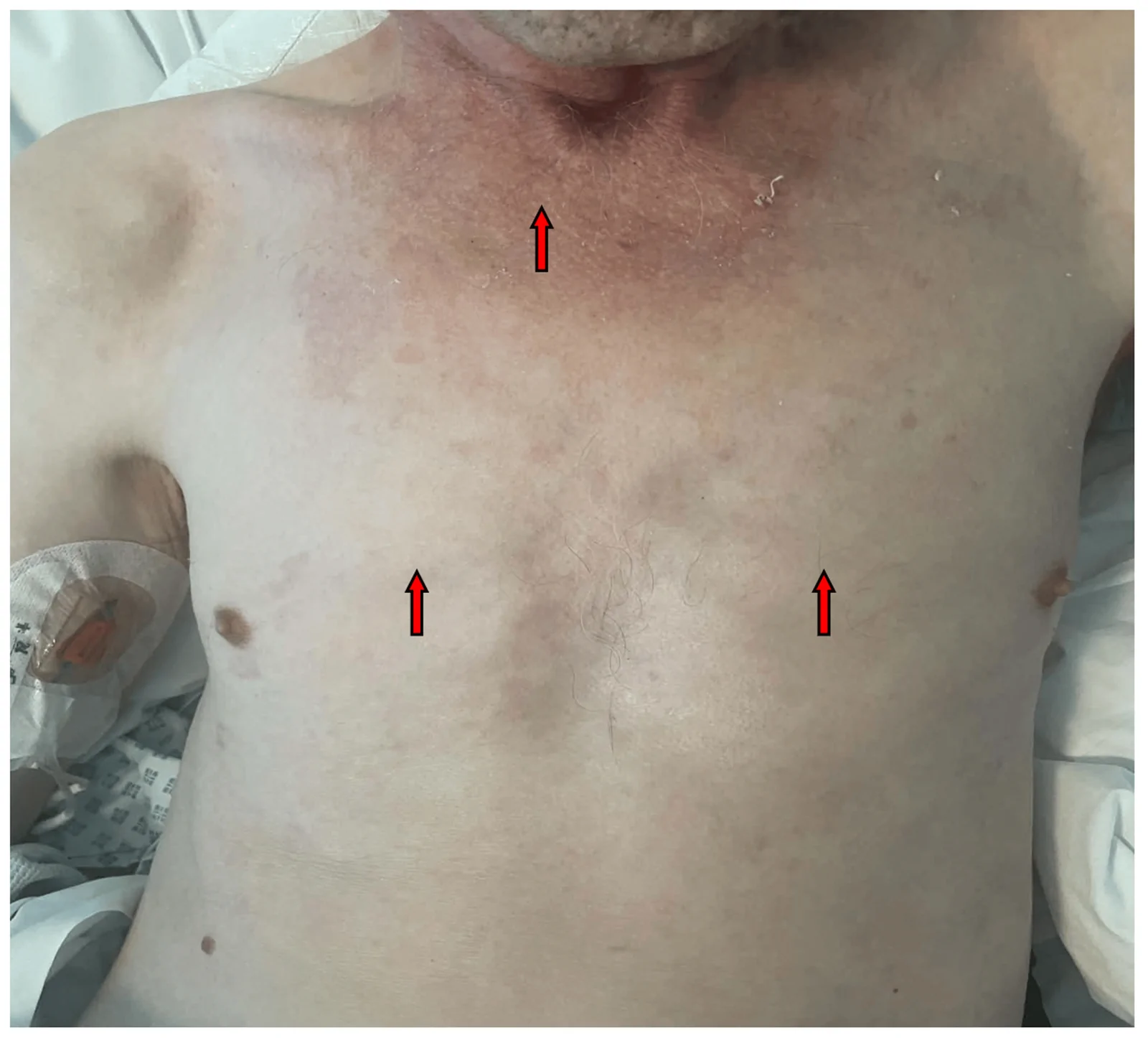
Cutaneous lesions demonstrated near-complete resolution following immunosuppressive therapy.

## Discussion

Late-onset systemic lupus erythematosus (SLE), defined as disease onset after 50 years of age, represents approximately 10-20% of all SLE cases and is increasingly recognised as a distinct clinical phenotype with unique immunological and clinical characteristics compared with early-onset disease [2,3]. Although constitutional symptoms, serositis and pulmonary involvement are relatively common, classical manifestations such as malar rash, photosensitivity and lupus nephritis occur less frequently, often leading to delayed diagnosis because the presentation may mimic infection, malignancy or other age-related conditions [2,3]. Recent studies have challenged the traditional perception that late-onset SLE is a benign disease, demonstrating that patients may accumulate substantial organ damage despite lower inflammatory disease activity owing to diagnostic delay, comorbidities and complications associated with ageing.

Male SLE remains particularly uncommon, accounting for fewer than 10% of all SLE cases, and frequently presents with atypical manifestations that contribute to diagnostic delay [1]. Compared with female patients, men are more likely to develop greater cumulative organ damage and poorer long-term outcomes, making a high index of clinical suspicion essential when evaluating older men with unexplained multisystem inflammatory disease [1].

Our patient's presentation illustrates these diagnostic challenges. Rather than presenting with classical rheumatological manifestations, he initially developed upper gastrointestinal bleeding associated with severe oesophageal candidiasis requiring intensive care admission. This unusual presentation prompted extensive investigation for HIV infection, viral illnesses and occult malignancy before an autoimmune disorder was considered. Opportunistic fungal infection is uncommon at the initial presentation of SLE and is more frequently encountered following immunosuppressive therapy. However, severe fungal infections may complicate the clinical course of patients with SLE and contribute to significant morbidity.

An additional notable feature of this case was the patient's recent non-ST-elevation myocardial infarction (NSTEMI). Cardiovascular disease remains one of the leading causes of morbidity and mortality in SLE and reflects the combined effects of chronic inflammation, endothelial dysfunction, accelerated atherosclerosis and thrombosis [5]. The presence of lupus anticoagulant in our patient raises the possibility that an autoimmune prothrombotic mechanism contributed to his acute coronary syndrome. Although antiphospholipid syndrome requires persistent antibody positivity together with compatible clinical criteria, lupus anticoagulant remains the strongest laboratory predictor of arterial thrombosis and warrants repeat testing together with long-term cardiovascular risk assessment [5]. Recent studies focusing specifically on late-onset SLE have further demonstrated that this subgroup may experience significant morbidity despite apparently lower disease activity, emphasising that late-onset disease should not be considered benign [6].

Although infections are well-recognised complications of established SLE, they usually occur following prolonged corticosteroid exposure or treatment with immunosuppressive agents. Opportunistic fungal infections preceding the diagnosis of SLE are considerably less common and may obscure the underlying autoimmune disease. Abnormalities in innate and adaptive immunity, including complement pathway dysfunction, impaired neutrophil activity and dysregulated immune responses, may contribute to increased susceptibility to opportunistic pathogens even before immunosuppressive therapy is initiated. Consequently, severe Candida oesophagitis in an apparently immunocompetent adult should prompt careful evaluation for underlying disorders including HIV infection, haematological malignancy, diabetes mellitus and, less commonly, systemic autoimmune disease. In our patient, extensive investigations excluded these alternative causes, while the subsequent development of persistent cutaneous lesions, pleural effusions, urinary abnormalities and highly specific lupus serology established the diagnosis of multisystem SLE. Recent nationwide cohort data have demonstrated that fungal infections among hospitalised patients with SLE are associated with increased morbidity and healthcare burden, highlighting the importance of early recognition and appropriate management [7].

As the patient's illness evolved, persistent photodistributed cutaneous lesions, bilateral pleural effusions, microscopic haematuria, proteinuria, hypocomplementaemia, strongly positive antinuclear antibodies, high-titre anti-double-stranded DNA antibodies, positive extractable nuclear antigen antibodies and lupus anticoagulant positivity established the diagnosis of multisystem SLE. These findings fulfilled the 2019 European League Against Rheumatism/American College of Rheumatology (EULAR/ACR) classification criteria, which demonstrate excellent sensitivity and specificity for SLE classification [4]. Histopathological findings suggestive of subacute cutaneous lupus erythematosus further strengthened the diagnosis and were particularly valuable given the patient's atypical age, sex and clinical presentation.

Pulmonary involvement manifested as bilateral pleural effusions consistent with lupus serositis. Serositis is recognised as one of the more common manifestations of late-onset SLE and may be the dominant presenting feature, often prompting extensive investigations for infection, malignancy or cardiac disease before autoimmune causes are considered [2,3,6]. This case highlights the importance of integrating pulmonary, dermatological, renal and immunological findings when assessing unexplained multisystem inflammatory disease.

Renal involvement was suggested by persistent microscopic haematuria and proteinuria despite preserved renal function. Although severe proliferative lupus nephritis occurs less frequently in late-onset SLE than in younger patients, renal disease remains clinically important because delayed diagnosis may result in irreversible chronic kidney damage [2,3,6]. Careful longitudinal monitoring of renal function and urinary abnormalities is therefore essential even when renal impairment is initially mild.

Recent advances in immunological research have expanded understanding of SLE pathogenesis, including the role of B-cell abnormalities, immune dysregulation and altered immune tolerance mechanisms in disease development and heterogeneity [8]. Genomic studies have further identified pathways involving interferon signalling, immune regulation and genetic susceptibility that contribute to variable clinical expression of SLE [9].

Systematic analyses comparing late- and early-onset lupus phenotypes have highlighted important differences in clinical manifestations, laboratory features and patterns of organ involvement, supporting the concept that late-onset SLE represents a distinct clinical subgroup rather than simply delayed presentation of typical disease [10].

Management represented a significant therapeutic challenge because active multisystem autoimmune disease required prompt immunosuppression while recent severe oesophageal candidiasis increased the risk of further opportunistic infection. Current EULAR recommendations advocate early diagnosis, prompt control of disease activity, minimisation of glucocorticoid exposure and careful monitoring for infectious complications during immunosuppressive therapy [11,12]. Treatment should be individualised according to organ involvement, comorbidities and infection risk, with multidisciplinary collaboration central to achieving optimal outcomes.

In our patient, close collaboration between intensive care, infectious diseases, rheumatology, gastroenterology, dermatology, nephrology and respiratory medicine enabled appropriate antifungal therapy to be balanced safely with immunosuppression. The favourable clinical response following high-dose corticosteroids and hydroxychloroquine therapy highlights the importance of coordinated multidisciplinary care in complex presentations.

This case highlights an unusual presentation of late-onset male systemic lupus erythematosus that coexisted with severe oesophageal candidiasis, gastrointestinal bleeding with haemodynamic instability that required intensive care unit admission before the development of more recognisable multisystem autoimmune features. Histologically suspected subacute cutaneous lupus erythematosus, bilateral pleural effusions, urinary abnormalities and highly specific lupus serology ultimately established the diagnosis according to the 2019 EULAR/ACR classification criteria [4]. The coexistence of recent NSTEMI and lupus anticoagulant positivity further suggests a possible autoimmune-mediated prothrombotic mechanism contributing to cardiovascular disease [5]. This case also emphasises the importance of recognising diagnostic delay as a major challenge in SLE, particularly among older men presenting with atypical manifestations [1,10,13]. Clinicians should maintain a high index of suspicion for occult autoimmune disease when opportunistic infection, dermatological manifestations, pulmonary serositis and renal abnormalities coexist despite negative investigations for infection and malignancy.

## Conclusions

Late-onset SLE in males is rare and often presents with atypical multisystem disease, leading to diagnostic delay. This case demonstrates occult SLE presenting in the ICU with esophageal candidiasis, pleural effusions, renal involvement, cutaneous manifestations suggestive of subacute cutaneous lupus erythematosus (SCLE), and possible autoimmune-associated cardiovascular disease. Clinicians should maintain a high index of suspicion for SLE in patients with unexplained multisystem inflammatory disease, particularly when dermatological, pulmonary, and renal manifestations coexist. The presence of severe opportunistic infection, unexplained serositis, persistent cutaneous lesions, and positive autoimmune serology should prompt consideration of an underlying systemic autoimmune disorder, even in older male patients who do not fit the typical demographic profile of lupus. This case also highlights the importance of multidisciplinary collaboration in balancing immunosuppression against infectious risk in critically ill patients, emphasising that early recognition, timely initiation of appropriate therapy, and coordinated specialist input are essential to improve outcomes and minimise irreversible organ damage.
